# Barriers to Post-Mastectomy Breast Reconstruction: A Comprehensive Retrospective Study

**DOI:** 10.3390/cancers17122002

**Published:** 2025-06-16

**Authors:** Kella L. Vangsness, Ronald M. Cornely, Andre-Philippe Sam, Naikhoba C. O. Munabi, Michael Chu, Mouchammed Agko, Jeff Chang, Antoine L. Carre

**Affiliations:** 1 Community Memorial Hospital System, Ventura, CA 93036, USA; 2 Emory University, Atlanta, GA 30322, USA; 3 Riverside School of Medicine, University of California, Riverside, CA 92507, USA; 4 Department of Plastic and Reconstructive Surgery, Columbia University, New York, NY 10032, USA; 5 Department of Plastic & Reconstructive Surgery, Kaiser Permanente, Los Angeles, CA 90034, USA; 6 Department of Plastic & Reconstructive Surgery, City of Hope, Duarte, CA 90034, USA

**Keywords:** breast reconstruction, barriers, mastectomy, plastic surgery, breast cancer

## Abstract

Background and Objectives: Breast reconstruction following mastectomy improves quality of life and psychosocial outcomes, yet it is not consistently performed despite multiple federal mandates. Current data shows decreased reconstruction in minority races, those with a low socioeconomic status, and those holding public health insurance. Many barriers remain misunderstood or unstudied. This study examines barriers to post-mastectomy breast reconstruction to promote a supportive clinical climate by addressing multifactorial obstacles to equitable access to care. Materials and Methods: The California Cancer Registry Data Surveillance, Epidemiology, and End Results (SEER) database and California Health and Human Services Agency Cancer Surgeries Database (2013–2021 and 2000–2021, respectively) were used in this retrospective observational study on mastectomy with immediate breast reconstruction (IBR), delayed breast reconstruction (DBR), or mastectomy only (MO) rates. Data were collected on age, sex, race, insurance type, hospital type, socioeconomic status, and residence. Pearson’s chi-square analysis was performed. Results: We found that 168,494 mastectomy and reconstruction surgeries were performed (82.36% MO, 7% IBR, 10.6% DBR). The 40–49 age group received significantly less MO (38.1%) compared to the 70–74 age group (94.8%, (*p* = <0.001). Significantly more reconstruction was carried out in patients with private, HMO, or PPO insurance (IBR 75.86%, DBR 75.32%, *p* = <0.001). Almost all breast surgeries were in urban areas as opposed to rural/isolated rural areas (96.02% vs. 1.55%, *p* = <0.001). There was no significant difference between races. Of all surgeries, 7.46% were completed in a cancer center with significantly higher rates of IBR. LA County, San Luis Obispo/Ventura County, and Northern CA had significantly more MO than other regions (*p* = <0.001). Conclusions: Reconstruction rates after mastectomy are low, with only 17.64% of patients undergoing reconstruction. Nationally, 70.5% of patients received MO, with 29.6% undergoing reconstruction. Significant factors positively contributing to reconstruction were private insurance, high SES, cancer center care, and urban residency. Identified barriers include public health insurance enrollment, rural or non-urban residence, older age, low SES, and non-white race/ethnicity, indicating potential monetary influences on care.

## Simple Summary

Many demographics affect post-mastectomy reconstruction care, including an older age, lower income, care at a public hospital, rural care, and non-White race. This data is significant because it identifies vulnerable populations and factors impeding breast reconstruction despite multiple federal regulations in place.

## 1. Introduction

Breast reconstruction for post-mastectomy cancer patients is not consistently performed as part of routine care despite a 13% average lifetime risk of breast cancer (one in eight women [1]), and proven psychosocial, physical, and quality-of-life benefits [2,3,4,5]. Current surgical breast cancer options include removal of the whole breast or a portion of it, with improved quality of life and major psychological benefits if reconstructive efforts are undertaken [6]. Reconstructive options include immediate breast reconstruction (IBR) with autologous tissue replacement or implant-based interventions, or delayed breast reconstruction (DBR), which takes place further along in the treatment course. IBR is associated with fewer operations and potential improved psychosocial benefits [3], but carries higher complication rates compared to DBR. Radiation therapy [3] and tumor characteristics [7] are major determinants in IBR, both of which significantly reduce reconstructive rates.

Between 1998 and 2015, only 5.2% of patients who underwent a mastectomy would go on to receive IBR [8]. In attempts to improve outcomes, the Women’s Health and Cancer Rights Act of 1997 [9] was instituted to require coverage for breast reconstruction following mastectomy. It stipulated that coverage was to include all stages of reconstruction to match breasts in shape, size, and form, as well as prostheses and procedural complications. The Patient Protection and Affordable Care Act of 2010 [10] required that from then on all women undergoing mastectomy would be referred to a plastic surgeon [10]. This continues to be supported by the American College of Surgeons and National Accreditation Program for Breast Cancer. Specific religious sponsored health plans and Medicaid do not cover costs; however, Medicare may cover reconstructive procedures if deemed medically necessary by the surgeon. Rates have since improved, and as of 2013, the rate of all reconstruction types was 17% nationally [11], with states varying between 17% and 35% [3,12,13,14]. Over this same time period, IBR rates grew as the desired reconstruction option from 6.3% in 1996 to 16.8% in 2015 [8].

Reports vary [15], but they indicate that DBR may impact body image and reduce overall quality of life [3,16] when compared to immediate reconstruction [8]. Delayed or absent reconstruction may increase physical health risks such as chronic pain and long-term musculoskeletal strain from uneven weight distribution following unilateral mastectomy [2]. These physical and psychological challenges are further exacerbated by socioeconomic and healthcare disparities, which disproportionately impact underserved populations [17,18].

Negatively contributing factors in the current literature are identified as a minority race and ethnicity [6,19,20], geography, public health insurance type [19,21], income <USD 38,000 a year [19], hospital type [22], older age, increased number of comorbidities [23], treatment type [7,24], and patient lack of awareness of options [7] or preference to forgo or delay reconstruction [25].

Statistically significant findings support racial disparities in breast reconstruction [6,26,27]. White, Non-Hispanic White, and Caucasian patients have been found to be statistically significantly more likely to receive mastectomies [14,28] when compared to African American [29], Asian [28,29], Latina [6,19,20], American Indian, and Alaska Native patients [14]. African American women are also less likely to receive breast reconstruction than Caucasian and Asian women [14,24], while Alaska Natives are the least likely to receive breast reconstruction out of all racial groups, with higher rates of treatment delays [14].

Geographic barriers have also been observed when comparing the eastern US region to the western and southern regions, the relative distances to surgery centers, the plastic surgeon density, and the presence of a non-academic or cancer center [6,26,27].

Additionally, patient preferences for a reconstructive approach may influence reconstruction rates. Bailey et al. found that amongst 134 patients who underwent MO procedures, 38 (28%) declined a plastic surgeon referral, 37 (28%) were not IBR candidates, and 29 (22%) underwent delayed reconstruction [25]. An additional 21 (16%) were seen by a plastic surgeon but declined reconstruction, 3 (2%) were referred but did not attend their appointment, and 6 (4%) had unknown reasons for not undergoing reconstruction [25]. The type of breast cancer treatment also contributes to reconstruction decisions [30]. Notably, adjuvant radiation therapy has been identified as a significant predictor of breast reconstruction among Caucasian women, but not among African American women [18,31].

Despite the available literature outlining barriers to post-mastectomy breast reconstruction, much of the available data is limited, with most focused on racial disparities. Given the paucity of information regarding the impacts of social determinants of health on breast reconstruction, the present literature could benefit from a more comprehensive analysis. This study aims to provide an updated assessment of current barriers to accessing post-mastectomy breast reconstruction.

## 2. Materials and Methods

### 2.1. Data Sources

This retrospective descriptive study utilizes the California Cancer Registry Data Surveillance, Epidemiology, and End Results (SEER) database in conjunction with the California Health and Human Services Agency Cancer Surgeries Database (CHHS). The CHHS database was used to collect the number of cancer surgeries performed in California hospitals between 2013 and 2021. Categories included year, county of hospital location, hospital name, type of cancer surgery, and number of cancer surgeries performed in Californian acute care hospitals (ICD9 and ICD-10 codes). Hospitals were also identified and characterized as academic vs. community, private or public, or cancer centers using publicly available data. The SEER 22 “Incidence-SEER Research Data, 17 Registries, Nov 2023 Sub (2000–2021)” registry was used for its national, comprehensive, and cancer-specific population-based data. These databases were selected for their comprehensive population-level data and use in prior high-impact analyses of breast reconstruction barriers [3,20,21].

All data is de-identified within both databases; therefore, no informed consent was required.

### 2.2. Inclusion Criteria

Inclusion criteria were female patients with a diagnosis of breast cancer who underwent specific procedures during the years 2000–2021. Patient inclusion criteria were eligible patients undergoing breast implant insertion or replacement post-mastectomy (CPT 19340, 19342); the use of tissue expanders with or without implant replacement (CPT 19357, 11971, 11970); revision of the peri-implant capsule, including capsulotomy, capsulectomy, and complete capsulectomy (CPT 19370, 19371); or AlloDerm [LifeCell Corporation, Branchburg, USA] implantation in breast reconstruction (CPT 15777). Patients who received a lumpectomy or breast-conserving surgery were excluded from this study. Collected data included the patient ID, race/ethnicity, age, sex, comorbidities, income/median household income/SES, marital status at diagnosis, rural–urban continuum/county attributes, site-specific surgery, surgery of primary site, year of diagnosis, year of follow-up, first malignancy/sequence number, admission type, insurance type, cancer versus non-cancer center, American Joint Committee on Cancer staging system, time from diagnosis to treatment, and reporting source.

Site-specific surgery coding for the breast included IDC-0: 174.0–174.6, 174.8–174.9, 175.9. Surgery codes for the primary surgery site were categorized by the type of mastectomy or breast reconstruction. Mastectomy type: 20–24—Breast-conserving or -preserving surgery, 20—Partial mastectomy, less than total mastectomy, 40—Total (simple) mastectomy, 41—Simple bilateral mastectomy, 76—Bilateral mastectomy for single tumor, 43—Simple mastectomy with tissue expanders, 50—Modified radical mastectomy, 60—Radical mastectomy, 71—Extended radical mastectomy, 80—Mastectomy, 90—Breast surgery + pathological specimen, 41, 51, 61, 71—Without removal of uninvolved contralateral breast, 42, 52, 62, 72—With removal of uninvolved contralateral breast. Breast reconstruction types: 30—Subcutaneous mastectomy (nipple-sparing mastectomy), 43, 47, 53, 57, 64, 68—Reconstruction, 44, 48, 54, 58, 65, 69—Tissue, 45, 49, 55, 59, 66, 73—Implant, 46, 56, 63, 67, 74, 75—Combined tissue and implant. Code 90—Death certificate only, unknown if surgery performed.

### 2.3. Statistical Analysis

The SEER database was used for a detailed analysis of demographics, initial surgery, and reconstruction type, if any. CHHS focused data allowed us to analyze state trends by hospital type, county, and breast surgery type. California’s vast socioeconomic landscape allowed for an in-depth view of disparities specific to geography, institutions, and patients. Together, these sources allowed for a robust examination.

Descriptive statistics we analyzed included frequencies and percentages (i.e., distribution) of breast cancer surgeries by year, county, and hospital. Descriptive statistics of the number of surgeries by hospital characteristics were analyzed by stratifying the mean number of surgeries by the categories of interest. An independent sample *t*-test was applied to determine whether mean differences were statistically significant.

Surgery codes were categorized into three groups: mastectomy only (MO), immediate breast reconstruction (IBR), or delayed breast reconstruction (DBR). Complications were identified as the removal of an intact implant due to reported infection, malposition, capsular contraction (CPT 19328), or ruptured implant (CPT 19330). Data were analyzed using a chi-square test of independence to understand if there were demographic differences between patients across groups. Pearson’s chi-squared analysis was performed. A *p*-value of <0.05 was deemed to be significant.

For age, a mean difference analysis was performed using a one-way analysis of variance to understand if the average ages of patients were statistically significantly different from one another. Age was additionally analyzed by category. Pearson’s chi-square test of independence was employed to search for statistically significant differences amongst categorical variables across the three surgical groups, to evaluate if differences occurred by chance.

Descriptive statistics, chi-squared tests, and *t*-tests were employed where appropriate, and age comparisons were made using a one-way ANOVA aligning with the standard epidemiological methodology and previous retrospective cohort studies. Descriptive and bivariate analyses were chosen to avoid overinterpreting causality or misrepresenting associations without linked data.

Prior work by Lang et al. demonstrates the SEER database’s utility in tracking trends and disparities in care, while published work by Sergesketter et al. validates the added benefit of tracking sociodemographic trends utilizing this database [3,20]. SEER-specific characteristics are essential to allow for the analysis of trends over time, particularly in the context of federal and state mandates on breast reconstruction post-mastectomy. Utilizing sampling data would significantly limit the ability to make conclusions on national reconstruction trends. Albornoz et al. and Sergesketter et al. also demonstrate the utility of SEER in identifying geographic and sociodemographic disparities, which is essential in the present analysis to allow the exploration of barriers to post-mastectomy care [20,21]. The success of prior studies in producing high-impact findings reinforces SEER’s reliability, especially given that it is specifically tailored to cancer epidemiology, which aligns with this study’s objectives.

## 3. Results

### 3.1. California Health and Human Services Agency Cancer Surgeries Database

California is diverse in almost all socioeconomic variables, which allow for the extrapolation of data for a comprehensive assessment of barriers. A total of 243,887 breast surgeries were performed in California between the years 2013 and 2021 in 322 hospitals. Of these surgeries, 168,494 were mastectomy-only, IBR, or DBR, which had rates of 82.36%, 7%, and 10.6%, respectively. Private hospitals comprised 83% (268) and public hospitals 17% (54) of the 322 hospitals examined. The surgeries at private hospitals accounted for 88% of the breast cancer surgeries performed, higher than the expected 83%. Public hospitals accounted for 12% of breast cancer surgeries, lower than the expected 17%. A statistically significant difference was found in the average number of surgeries performed in private (M = 804, SD = 1053.38) vs. public (M = 526, SD = 681.87) hospitals (t (111.13) = 2.46, *p* < 0.05; equal variances were not assumed).

Academic hospitals totaled 14% (44) and community hospitals 86% (278) of the 322 hospitals. The breast cancer surgeries performed at academic hospitals accounted for 28%, higher than the expected 14%. Public hospitals accounted for 72% of breast cancer surgeries, lower than the expected 86%. There was a statistically significant difference in the average number of surgeries performed in academic (M = 1548, SD = 1369.62) vs. community (M = 632, SD = 874.53) hospitals (t (48.70) = 4.30, *p* < 0.001; equal variances were not assumed).

Cancer centers comprised 2.5% (8) and non-cancer centers 97.5% (314) of the 322 hospitals included in the analysis. Cancer center surgeries accounted for 9.4% of all breast cancer surgeries performed, almost four times higher than the expected 2.5%. A statistically significant difference was found in the average number of surgeries performed in cancer centers (M = 2867, SD = 1427.81) vs. non-cancer centers (M = 704, SD = 934.99) (t (7.15) = 4.26, *p* < 0.01; equal variances were not assumed).

Between 2013 and 2021, 243,887 breast surgeries were performed in the state of California. The highest rate of all breast surgeries over this time period was in the counties of Los Angeles (*n* = 64,662, 26.51%), San Diego (pop. *n* = 23,399, 9.59%), and Orange (*n* = 22,007, 9.02%). The populations of these counties were roughly 33 million, 13 million, and 3 million, respectively.

No significant differences were found between racial groups and rates of breast reconstruction in the state of California. Cancer centers had statistically significantly higher rates of IBR (7.64%) compared to other hospital types. Most of the reported reconstruction surgeries in California were completed in urban areas (96.02%) compared to rural locations (1.55%). Only 0.99% of IBR occurred in rural areas. The highest frequency of IBR was in Orange County. The highest mastectomy-only rates occurred in Los Angeles County, San Luis/Obispo/Ventura County, and Northern California.

Many counties had a lower than 0.10% rate of total breast cancer procedures. These were found in the counties of Amador (pop. 40k, *n* = 33, 0.01%), Calaveras (pop. 45k, *n* = 64, 0.03%), Del Norte (pop. 27k, *n* = 75, 0.03%), Inyo (pop. 19k, *n* = 82, 0.03%), Lake (pop. 68k, *n* = 44, 0.02%), Lassen (pop. 31k, *n* = 3, 0.00%), Madera (156k, *n* = 167, 0.07%), Plumas (19.5k, *n* = 30, 0.01%), San Benito (64k, *n* = 85, 0.03%), Tehama (65k, *n* = 62, 0.03%), Trinity (pop. 135.k, *n* = 6, 0.00%), and Yuba (pop. 72.5k, *n* = 187, 0.08%).

### 3.2. Surveillance, Epidemiology, and End Results (SEER) Database

SEER-specific database characteristics are essential to the analysis of barriers. Nationally, 29.6% received post-mastectomy breast reconstruction. Within this group that underwent post-mastectomy breast reconstruction, 26.4% received IBR, 3.2% underwent DBR, and 70.5% had MO. Individuals of non-white race/ethnicity had a decreased likelihood of undergoing breast reconstruction. Those who identified as non-Hispanic White individuals were more likely to receive IBR, at a rate of 27.7% (68.7% MO, 27.7% DBR). Hispanic, Black, Asian or Pacific Islander, and American Indian/Alaska Native patients received MO at rates under 80% (73.3%, 74.3%, 75.5%, and 77.1%, respectively).

**Figure 1:**
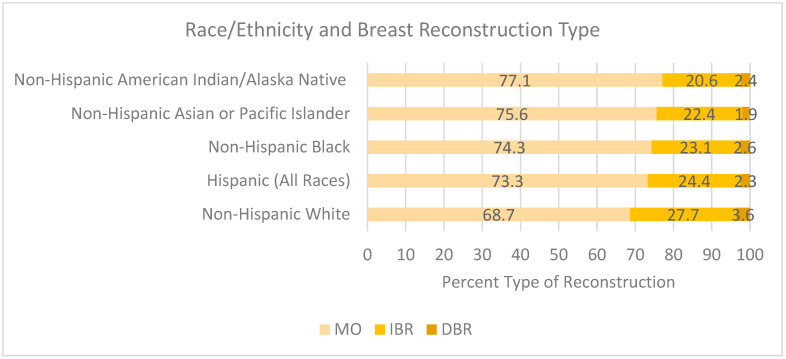
Race/ethnicity and breast reconstruction type.

Of those who received breast reconstruction, the majority underwent IBR as opposed to DBR: Hispanic: 24.4% IBR vs. 2.3% DBR, Black: 23.1% IBR vs. 2.6% DBR, Asian or Pacific Islander: 22.4% IBR vs. 1.9% DBR, and American Indian/Alaska Native: 20.6% IBR vs. 2.4% DBR (Figure 1).

When examining the state of California within the SEER database specifically, we found that only 17.64% of patients proceeded with any reconstruction after mastectomy, and 82.4% underwent an MO procedure. Of those who underwent reconstruction, 10.6% received DBR and 7% IBR. The reconstruction rate of the 40–49-year-old age group was greater than that of the 70–74-year-old age group and demonstrated a statistically significant difference. However, the 40–49-year-old age group underwent mastectomy only at a rate of 38.1%, compared to 94.8% of those aged 70–74 (*p* = <0.001).

**Figure 2:**
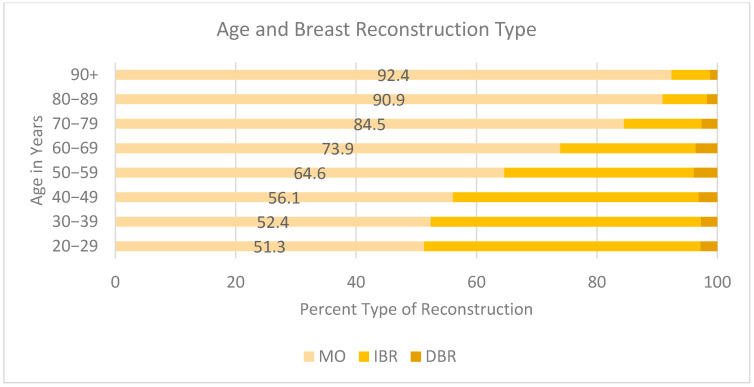
Age and breast reconstruction type.

Decreased rates of reconstruction were found in older age (Figure 2). The highest rates of MO were in the 90+ (92.4%), 80–89 (90.9%), 70–79 (84.5%), 60–69 (73.9%), and 50–59 (64.9%) age groups compared to the 20–29 (51.3%), 30–39 (52.4%), and 40–49 (56.1%) age groups. Younger patients were more likely to opt for IBR versus DBR if they did undergo breast reconstruction. Those aged 20–29 underwent IBR at a rate of 45.9% vs. 2.8% DBR, 30–39 IBR 44.9% vs. 2.7%, 40–49 40.8% vs. 3.1%. Rates of IBR decreased as age increased, dropping by almost half between the 70–79 and 80–89 age groups (12.9% and 7.4%, respectively). The group with the lowest rate of any reconstruction type was the 90+-year-old cohort, with 6.4% IBR and 1.2% DBR.

**Figure 3:**
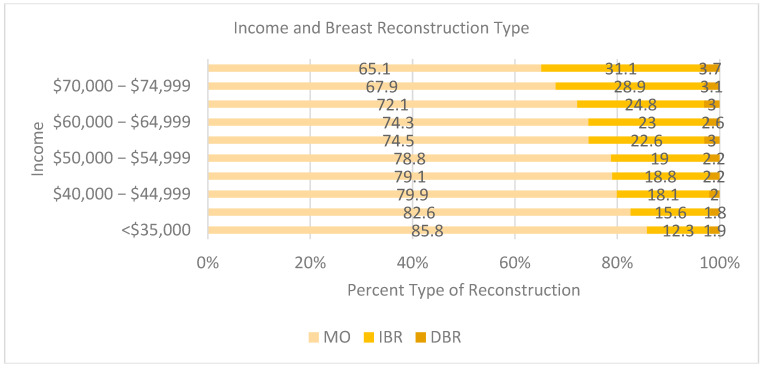
Income and breast reconstruction type.

An income of <USD 70,000 was associated with a decreased likelihood of receiving breast reconstruction (Figure 3). As income increased, so did reconstruction rates for both IBR and DBR. Those earning less than USD 40,000 a year had above 80% MO rates [USD <35k (85.8 % MO, 12.3 % IBR, 1.9 % DBR); USD 35–29k (82.60% MO, 15.6% IBR, 1.8% DBR)]. Between USD 40,000 and USD 64,999, MO rates were between 79.9% and 72.1% and IBR between 18.1% and 24.8%. Once income was greater than USD 70,000, the rate of MO dropped below 70%, and at USD 75,000, IBR was above 31% [USD 70,000–74,999: 67.9% MO, 28.9% IBR, 3.1% DBR; and USD >75k: 65.1% MO, 31.1% IBR, 3.7% DBR].

The insurance type was also examined as a variable. We found that 8% of all MO patients were on public health insurance. In the years examined, 75% of IBR that was performed was covered under private insurance. Significantly more reconstruction was carried out in patients who had private, HMO, or PPO insurance, at rates of IBR at 75.86% and DBR at 75.32% (*p* = <0.001), compared to those with government insurance for the elderly or low earning specifically.

**Figure 4:**
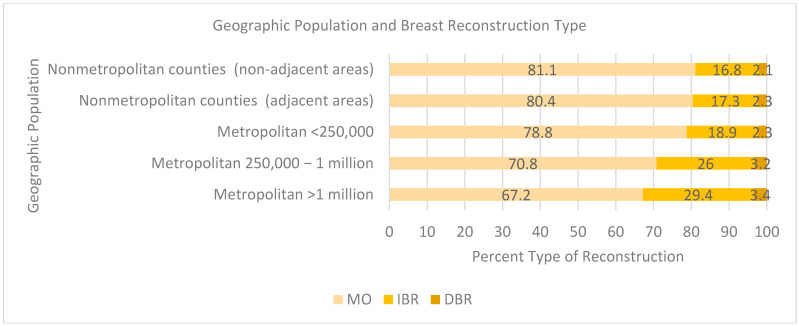
Geographic location population and breast reconstruction type.

Those whose residence was outside of an urban population (<1 million) were less likely to receive breast reconstruction, with an MO reconstruction rate greater than 70% (Figure 4) (81.1% not adjacent to a metropolitan area, 80.4% adjacent to a metropolitan area, 78.8% <250,000 population, 70.8% 250,000 to 1 million population). At a population density of 1 million, the proportion of IBR cases was approximately 30%. However, there were minimal differences between the area of residence and percentage of DBR (3.4% per million people versus 2.1%) when not adjacent to a metropolitan area.

## 4. Discussion

This work has found that the national breast reconstruction rate is currently 29.6%, which is an increase from 11.7% in 1996 and 21.7% in 2008 [3]. Nonetheless, the rate remains relatively stagnant despite multiple federal mandates put into place. This study corroborates previous findings [27] and provides an update on current barriers, which include the insurance type (private vs. public), hospital type, geographic residence, socioeconomic status (SES), and age, as potential areas of future mandate improvements.

Discordant racial data was found when comparing national findings with those specific to California. While national data demonstrates significant racial disparities, no differences were observed in the California-based analysis. Nationally, racial and ethnic minorities experience inequitable access to breast reconstruction due to sociodemographic disparities and implicit biases [30,32]. This study shows that non-Hispanic White women are more likely to undergo mastectomy with immediate breast reconstruction (IBR) than Hispanic, Black, Asian or Pacific Islander, and American Indian/Alaska Native women. Our results are congruent with previous findings by Sergesketter et al. [20], who found racial and ethnically marginalized groups (non-Hispanic Black and Hispanic women) to have a statistically significantly lower likelihoods of undergoing reconstruction post-mastectomy in a national cohort [17]. A systematic review conducted by Doren et al. [32] also found a higher likelihood of undergoing overall reconstruction in those who identified as White compared to Black patients [32].

However, California-specific analyses suggests that the SES, insurance type, and geographic location may play more prominent roles in determining access to breast reconstruction than race. These findings align with the current literature and emphasize the complex interplay of system, physician, and patient-related factors contributing to disparities [33]. The observed disparity in California may be attributable to the state’s large and diverse population, varying socioeconomic conditions, and access to healthcare. From this data, we infer that areas with a larger and more diverse population may be better equipped to provide resources that allow for overcoming certain barriers, as demonstrated by race not being included as a barrier in California versus nationally.

Schafer et al. assessed the impact of the Affordable Care Act on racial and ethnic diversity between the years 2005 and 2022 [34]. Prior to federal mandate changes, American Indian or Alaska Native, Asian, and Black or African American individuals underwent less IBR compared to White patients (*p* < 0.001). Hispanic-identifying patients specifically obtained IBR at 28% compared to their White counterparts at 33.4% (*p* < 0.001). In the coming years, the trends did not equalize, and all non-White patients underwent IBR at a lower rate than that of White patients (*p* < 0.001), with Hispanics as the only exception, for whom the IBR rate increased to 53.8% compared to White patients at 47.9% (*p* < 0.001) [35]. Wirth et al. [35] identified persistent racial disparities nationally, particularly for Black women, which was not statistically significant in our California-focused analysis, suggesting that regional healthcare systems may moderate racial disparities. These findings highlight the need for region-specific and demographically informed interventions to improve equitable access to post-mastectomy breast reconstruction.

Patient-specific factors related to SES (e.g., income, occupation, education, and social and cultural capital) were also strongly associated with the variation in reconstruction rates, which supports a more nuanced understanding of access inequity. A higher SES correlated with higher breast reconstruction rates (30% of patients in high-SES groups vs. 10.03% in low-SES groups). Restrepo et al. also identified being White, having a higher income, and having a higher educational background as significant predictors of reconstruction rates, supporting the current study’s findings [19]. The insurance type also emerged as a key determinant. Private insurance (HMO/PPO) was associated with significantly higher rates of IBR and DBR compared to public health insurance, suggesting that federal policies—the Cancer Rights Act of 1998 and the Affordable Care Act of 2010—have been ineffective in addressing the reconstructive needs of breast cancer patients.

The geographic location played a major role, with significantly higher reconstruction rates in urban (96.02%) than rural areas (1.55%). Private hospitals had higher reconstructive surgery volumes than public hospitals, with private hospitals performing 88% of reconstructions, versus the expected 83%, and public hospitals performing 12%, versus the expected 17%. The statistically significant differences in reconstruction rates between hospital types support the idea that resource allocation and geographic factors influence the access to reconstructive care. The geographic isolation of rural communities and the relative concentration of hospital resources in urban areas exacerbate these access barriers. The availability of plastic surgeons and specialized facilities (e.g., cancer centers and hospitals) is critical to bridging the equity gap. Wareham et al. found that not only are there fewer breast surgeons than plastic surgeons but also that 14% of breast surgeons did not have a plastic surgeon within a 10-mile distance, though that is a fall from 25% in 2018 [36].

Cancer centers also demonstrated greater surgical volumes (mean = 2867 procedures per institution vs. 704 at non-cancer centers) and significantly higher IBR rates. Despite representing only 2.5% of hospitals in California, cancer centers performed a disproportionately high number of reconstructions and had significantly higher IBR rates (7.64%). Access to specialized care centers may facilitate equitable care for this complex patient population and underscores the critical role of cancer center care.

These findings are consistent with the prior literature, as Albornoz et al. [21] found that reconstruction rates are positively correlated with private hospital systems as well as private insurance carriers. The protective effects of a high SES and private insurance emphasize the role of economic factors in healthcare access, particularly when examined within California.

Older age is also negatively correlated to breast reconstruction of both types, consistent with findings by Cortina et al. [6]. Women are more likely to receive IBR at younger ages, with those aged 70+ receiving the majority of MO. However, likely confounding variables include an increased number of comorbidities, patient education, and personal choice, so limited conclusions can be made.

This study found that 17.64% of patients underwent any type of reconstruction following mastectomy. When comparing delayed versus immediate reconstruction, we found that 10.6% chose delayed and 7% underwent immediate reconstruction. This may provide insight into patient preference, but due to the lack of subjective data, no true causation can be highlighted. However, in a systematic review by Doren et al., the contributors to poor reconstruction rates included issues with individual or community interactions with the healthcare system (54% and 36%, respectively), the sociocultural environment (39%), and individual behavioral factors (31%) [32], allowing some inference to be made, but more objective data and analyses are required. Unmeasured and unexamined barriers remain, such as cultural perceptions, communication gaps, and implicit biases during clinical interactions, which could contribute to the observed disparities in reconstructive access.

Despite a comprehensive analysis, limitations remain. Due to California’s unique demographic and healthcare structure, data cannot be generalized to all national regions. Additionally, the absence of qualitative data prevented the exploration of patient-level factors that may influence decision-making, including but not limited to cultural attitudes or provider–patient communication and education. Future studies should integrate patient-reported outcomes, comorbidities, or a mixed-methods approach to overcome our lack of qualitative insights, as noted in the prior literature by Retrouvey et al. and Connors et al. [7,18]. An underrepresentation of rural patients and public hospitals may also skew data, potentially misrepresenting populations and resources. Although the cancer center data is compelling, it may not fully reflect the outcomes at non-specialized facilities where differences in surgeon expertise and available resources can affect results. Furthermore, racial, ethnic, and age disparities may not fully capture nuanced subgroup differences or systemic inequities.

### Advocacy and Policy Recommendations

Clinicians, health systems, and governmental entities could consider actionable steps to address these inequities. These may include introducing equity-focused counseling to address health literacy gaps, particularly among historically underserved populations; utilizing interpreters and providing culturally appropriate materials in a range of languages for improved communication and education; ensuring providers’ and staffs’ continued participation in implicit bias training; standardizing referral protocols based on pre-determined clinical criteria to minimize variability and potential bias in access to plastic surgery consultations; and implementing data-driven interventions by collecting patient-reported information on barriers such as cultural beliefs, mistrust, and financial limitations. These data could inform more tailored support services.

Health systems can play a key role by expanding outreach efforts, redistributing care services, and tracking equity metrics. A recent study by Stankowski et al. found that women with public health insurance and living furthest from a plastic surgeon were less likely to receive reconstruction [37]. Outreach to rural and underserved communities may include establishing mobile health units, satellite clinics, or telemedicine programs staffed by reconstructive specialists. Facilitating the distribution of plastic surgeons within breast cancer centers can also improve the access to timely, integrated care specifically targeting these barriers. Furthermore, tracking reconstructive procedure rates alongside race, ethnicity, insurance status, and geographic data can help systems identify inequitable gaps in care and set measurable goals for improvement.

Government institutions can support equity through policy and funding mechanisms. This includes incentivizing medical practice in underserved areas through loan forgiveness, grants, or reimbursement bonuses for reconstructive plastic surgeons. Operational funding could be directed toward non-urban hospitals to strengthen the reconstructive service infrastructure. In addition, investing in patient navigation programs, transportation assistance, and educational initiatives could help address logistical and informational barriers to care. Policy reforms mandating parity in insurance coverage for reconstruction and streamlining the authorization process may further reduce delays and denials. Lastly, regional collaboration could be promoted by fostering partnerships between high-volume cancer centers and smaller hospitals, improving referral networks and visiting specialists.

By implementing these coordinated strategies, clinicians, health systems, and governmental bodies can work in tandem to reduce disparities and ensure more equitable access to post-mastectomy breast reconstruction for all patients.

Future work could include multi-state studies to validate these findings and examine whether California’s lack of racial disparities is replicable elsewhere. This is best carried out with linked or enriched datasets, allowing researchers to perform multivariate analyses, if possible. Incorporating mixed methods or qualitative elements would allow researchers to further investigate the factors influencing patient decision-making, particularly racial minorities, cultural perceptions, provider communication, and awareness of reconstruction options. The goal of identifying disparities in access allows for targeted interventions.

## 5. Conclusions

Improvements in reconstruction rates after mastectomy are slow, with only 17.64% proceeding with reconstruction. Nationally, 70.5% of patients receive MO and 29.6% undergo reconstruction. Significant factors positively contributing to reconstruction are private insurance, a high SES, cancer center care, and urban residencies. Identified barriers include public health insurance enrollment, rural or non-urban residence, older age, a low SES, and non-white race/ethnicity, possibly indicating monetary influences on care.

## Data Availability

The original contributions presented in this study are included in the article. Further inquiries can be directed to the corresponding author.
